# The Influence of Drying upon Various Bacteria

**Published:** 1909-06-19

**Authors:** 


### Pathology.

### THE INFLUENCE OF DRYING UPON VARIOUS BACTERIA.

The results of careful and elaborate experiments made by G. G. Buckley on the resistance of some pathogenic micro-organisms to drying are interesting. The varieties used were staphylococcus pyogenes aureus, bacillus coli communis, bacillus typhosus, bacillus diphtheriæ, bacillus pestis, and spirillum choleræ. Of these staphylococcus pyogenes aureus was found to be the most, and spirillum choleræ the least, resistant to drying.

The latter cannot live in a condition of complete dryness. Of the other four, B. coli communis and B. typhosus proved more resistant than the B. diphtheriæ and B. pestis. The remaining conclusions may be quoted verbatim:—

Some organisms live longer in a moist and others in a dry atmosphere. In the first class are the spirillum choleræ and the bacillus coli communis, which live very much longer, and the bacillus typhosus and the bacillus pestis, which live only slightly longer in a moist atmosphere than in a dry one. In the second class are the staphylococcus pyogenes aureus and the bacillus diphtheriæ.

Speaking generally, the absolutely dry atmosphere of the desiccator is less harmful to the bacteria used in these experiments than the partially dry atmosphere of the room. This is possibly due, as has been suggested, to the rapid desiccation of the outer portions of the individual bacilli forming a complete protective coat for each organism. The cholera spirillum is an exception to this rule.

The material infected exerts a considerable influence on the powers of resistance to drying possessed by the different organisms; but this influence is not of the same kind on all bacteria nor under all conditions of dryness or moisture. The longest life was usually reached on plaster and lime-wood. The single exception was in the case of the bacillus pestis, which was very short-lived on lime-wood, and this was the case in each of ten series of experiments. All the organisms were short-lived on paper. As would be expected from the inability of the emulsion to sink into glass, and its consequent rapid drying, the organisms did not live very long on that material.

The effect of pine-wood was variable, and especially so in the moist chamber, pointing to the fact that some constituent or constituents of the wood were capable of acting injuriously upon the organisms in the presence of moisture. In all cases this variety of wood exercised an adverse influence on the organisms, and this suggests the advisability, from a sanitary standpoint, of the use of pine-wood, as far as possible, in such buildings as hospitals—and especially hospitals for infectious diseases.

Infection can persist in dry buildings, cloths, etc., for at least the following periods:—

| | |
|---|---|
| Staphylococcus pyogenes aureus ... | for 140 days. |
| Bacillus diphtheriæ ... ... ... | „ 114 „ |
| Bacillus coli communis ... ... | „ 92 „ |
| Bacillus typhosus ... ... ... | „ 91 „ |
| Bacillus pestis ... ... ... | „ 34 „ |
| Spirillum choleræ ... ... ... | „ 12 hours. |

These figures represent in each case the longest period during which the organism was found living on any material in the desiccator or in the air of the room.

In the case of certain organisms, infection may persist for even longer periods if the buildings, etc., are damp:—

| | |
|---|---|
| Bacillus coli communis ... ... | for 168 days. |
| Bacillus typhosus ... ... ... | „ 119 „ |
| Bacillus pestis ... ... ... | „ 45 „ |
| Spirillum choleræ ... ... ... | „ 21 „ |

